# Novel Contactless Sensors for Food, Beverage and Packaging Evaluation

**DOI:** 10.3390/s23198082

**Published:** 2023-09-26

**Authors:** Claudia Gonzalez Viejo, Damir D. Torrico, Sigfredo Fuentes

**Affiliations:** 1 Digital Agriculture Food and Wine, School of Agriculture, Food and Ecosystem Sciences, Faculty of Science, The University of Melbourne, Parkville, VIC 3010, Australia; sfuentes@unimelb.edu.au; 2 Faculty of Agriculture and Life Sciences, Lincoln University, Lincoln 7647, New Zealand; damir.torrico@lincoln.ac.nz; 3 Tecnologico de Monterrey, School of Engineering and Science, Ave. Eugenio Garza Sada 2501, Monterrey, NL 64849, Mexico

The use of traditional methods to evaluate food, beverages, and packaging tends to be time-consuming, labour-intensive, and usually involves high costs due to the need for expensive equipment, regular refill of consumables, skilled personnel and, in the case of sensory evaluation, incentives or payments involved for participants recruitment and/or panelists training and participation. Therefore, researchers have developed novel low-cost, rapid, and time-effective methods using digital and artificial intelligence technologies. This special issue (SI) focused on the novel methods developed using contactless sensors for food, beverages and packaging. This SI is composed of nine papers related to the use of spectral analysis using different methods, such as near-infrared spectroscopy to classify eggs into cage or free-range production practices [1], a semi-automatic low field nuclear magnetic resonance (LF-NMR) coupled with machine learning modelling to predict oxidation in edible oil [2], the use of vibrational spectroscopy to measure ethanol and methanol levels in pisco [3], and the use of an infrared laser sensor to monitor the gas-phase CO_2_ in Champagne headspace when swirling [4]. Other papers focus on the use of other specific sensors, such as a low-cost and portable electronic nose (e-nose) to predict aromas and roasting intensity in coffee [5] and the use of flexible sensors for monitoring oyster survival rates [6]. Furthermore, a computer vision method was presented using a conventional RGB camera coupled with machine learning modelling to predict the type of rice based on morphocolorimetric parameters obtained through the images [7]. On the other hand, two papers focused on the use of non-invasive biometrics (i) to assess self-reported responses as well as eye tracking and emotional responses towards specific regions of interest (ROI) in packaging and labels using contactless biometrics through the BioSensory© application (The University of Melbourne, Parkville, VIC, Australia) [8], and (ii) to evaluate the influence of label design and country of origin in wine labels on consumers conscious and subconscious responses using contactless biometrics also with the BioSensory© application [9].

Spectral analysis in foods and beverages is one of the most used methods to assess their chemical fingerprinting. This has been a very useful contactless tool for developing predictive models. Hoffman et al. [1] used a portable NIR device (950–1600 nm) to assess eggs chemical fingerprinting and used principal components analysis (PCA) and linear discriminant analysis (LDA) to analyze results. The authors successfully classified samples in cage and free-range production with an overall accuracy of 76% for whole eggs and 86% for egg whites and yolks separately. Osheter et al. [2] reported high accuracy (95%) on the classification of oxidation levels in edible oil using LF-NMR as inputs of convolutional neural networks (CNN) supervised machine learning modelling. In another publication, Menevseoglu et al. [3] used a Raman and Fourier-transform infrared spectroscopy handheld device to assess ethanol and methanol in Pisco through the bottle. Authors developed predictive models using partial least squares regression to predict ethanol and methanol using the spectral readings as inputs, obtaining correlation coefficient (R) values > 0.94. Lecasse et al. [4] used an infrared laser sensor to monitor CO_2_ release when swirling Champagne glasses. The authors found a sudden drop in CO_2_ contained in the headspace.

On the other hand, researchers have developed contactless sensors to assess food and beverage quality more efficiently, non-invasive, cost-effective and objectively. Gonzalez Viejo et al. [5] presented highly accurate artificial neural network models developed using a low-cost and portable e-nose output as inputs to classify coffee samples into their intensity levels with very high accuracy (98%) and to predict the peak area of specific aromatic volatile compounds, also with very high accuracy (R = 0.99). Liu et al. [6] developed flexible pressure sensors to monitor live oyster survival rates using an autoregressive integrated moving average (ARIMA) model, obtaining accuracies >79%.

Other contactless methods that have been developed as more efficient alternatives to traditional techniques were based on computer vision. Aznan et al. [7] presented a highly accurate method to classify rice into 15 different types using morphocolorimetric data obtained using RGB images and computer vision algorithms developed by the authors. The model was highly accurate (91%) with a deployment accuracy of 94% using independent samples.

Novel contactless methods have also been developed to assess consumers’ subconscious responses using biometrics elicited by packaging and labels. Fuentes et al. [8] successfully presented a prototype of an integrated method to evaluate independently different regions of interest (ROI) of labels using the BioSensory© application along with Gazepoint GP3 eye trackers (Gazepoint, Vancouver, BC, Canada) and software developed by the authors using the Affectiva software development kit (SDK; Affectiva, Boston, MA, USA) to assess emotional responses from consumers. Liu et al. [9] found an influence of the label information on consumers’ wine appreciation when tasting the same wine with different labels; the country of origin information was found to affect the perception. This was assessed using the BioSensory© application and Gazepoint GP3 eye trackers.

## Data Availability

Not applicable.
